# Mn-Fe Layered Double Hydroxide Intercalated with Ethylene-Diaminetetraacetate Anion: Synthesis and Removal of As(III) from Aqueous Solution around pH 2–11

**DOI:** 10.3390/ijerph17249341

**Published:** 2020-12-14

**Authors:** Guifeng Liu, Zongqiang Zhu, Ningning Zhao, Yali Fang, Yingying Gao, Yinian Zhu, Lihao Zhang

**Affiliations:** 1 Guangxi Key Laboratory of Environmental Pollution Control Theory and Technology, Guilin University of Technology, Guilin 541004, China; liuguifengmail@163.com (G.L.); 18835175975@163.com (N.Z.); fangyali0610@163.com (Y.F.); gyy88882020@163.com (Y.G.); lhzhang@glut.edu.cn (L.Z.); 2 State Key Laboratory of Environmental Aquatic Chemistry, Research Center for Eco-Environmental Sciences Chinese Academy of Sciences, Beijing 100085, China

**Keywords:** LDHs, EDTA, As, redox reaction, ion exchange

## Abstract

A novel adsorbent Mn-Fe layered double hydroxides intercalated with ethylenediaminete-traacetic (EDTA@MF-LDHs) was synthesized by a low saturation coprecipitation method. The behavior and mechanism of As(III) removed by EDTA@MF-LDHs were investigated in detail in comparison with the carbonate intercalated Mn-Fe layered double hydroxides (CO_3_@MF-LDHs). The results showed that EDTA@MF-LDHs had a higher removal efficiency for As(III) than As(V) with a broader pH range than CO_3_@MF-LDH. The large adsorption capacity of EDTA@MF-LDHs is related to its large interlayer spacing and the high affinity of its surface hydroxyl groups. The maximum adsorption capacity for As(III) is 66.76 mg/g at pH 7. The FT-IR and XPS characterization indicated that the removal mechanism of the As(III) on EDTA@MF-LDHs include surface complexation, redox, and ion exchange.

## 1. Introduction

Arsenic contamination is considered one of the most serious environmental issues. Many regions of the world are facing a certain degree of arsenic pollution [1,2,3]. The level of arsenic in many drinking water sources in Bangladesh and India is more than 20 times the standard limit (10 ppb) set by the World Health Organization (WHO) [4]. Arsenic pollution incidents of varying degrees have occurred in Guangxi, Henan, Gansu, Xinjiang, and Shandong provinces in China. Nearly 20 million people have been exposed to arsenic pollution, and nearly 580,000 square kilometers have been polluted by arsenic in the country [5]. Chile, Mexico, Argentina, Poland, Canada, Hungary, Japan, and other countries have also reported incidents of excessive arsenic concentration. About 200 million people are being exposed to excessive arsenic through consumption of contaminated drinking water according to the statistics from all over the world [6]. Studies have demonstrated that arsenic contaminated waterbodies have a significant potential to cause liver, lung, kidney, bladder, and skin cancer [7], and affect the intellectual development of children [8,9]. Among various technologies to solve the problem of arsenic contamination, adsorption, which is believed to be a simple, high efficiency, and low cost process, is the main method for the removal of arsenic. Most studies focus on the treatment of arsenic-containing wastewater under acidic conditions. However, in some cases, the arsenic-containing wastewater is strong alkaline such as the leachate wastewater discharged from the antimony refining process. For example, the pH of the raw water discharged from a smelter in Guangxi can be as high as 12.5. Nevertheless, there are only a few reports on the directly effective treatment of arsenic-containing wastewater under alkaline conditions. Due to the presence in an electrically neutral nonionic form (H_3_AsO_3_) in natural water [10], As(III) is generally reported to have low affinity to the surface of some adsorbents compared with As(V) [11,12,13]. Furthermore, valence has a great influence on the behavior and toxicity of arsenic [14,15]. Compared with As(V), As(III) has higher toxicity, solubility, and mobility, which can be absorbed faster in a biological system [16,17]. So it is desirable to develop an effective adsorbent for As(III) removal.

Recently, layered double hydroxides (LDHs) have been extensively studied owing to their advantage of large surface area, high anion adsorption/exchange capacity, structure controllability, and “memory effect” (The calcined product of LDHs, which is generally a mixed metal oxide, is exposed to water and anions under certain conditions, the structure of the layered LDHs can be restored) [18]. NMMB (Precipitation of Ni/Mn-LDHs onto pristine biochars) was prepared for As(V) removal [19], layered double hydroxide intercalated with MoS_4_^2−^ for oxoanions of As(III), As(V), and Cr(VI) uptake [20]; Mg-Al-Cl layered double hydroxide for simultaneous removal of Cu(II) and Cr(VI) [21]; edta·Mg-Al LDH for the uptake of Cu^2+^ and Cd^2+^ from an aqueous solution [22]. Currently, LDHs have been applied in practice; for example, the calcined Mn-Fe LDH was used for treatment of arsenic effluent and the study proved that calcined Mn-Fe LDH was an efficient adsorbent for arsenic effluent for 2 h [23]; the nanocrystallined Mg/Al LDHs was used for treatment of natural groundwater that contained arsenic and natural organic matter (NOM) [24]; the core-shell bio-ceramic/Zn-layered was used for phosphorus-containing municipal wastewater treatment [25]; and in the study of Jiang et al., high As(III) concentration in Bangladesh groundwater can be reduced to meet the national drinking water standards (<50 μg/L) for 2 g/L of Mg-Fe-Cl LDH [26].

The representative material of LDHs is Mg-Al hydrotalcite ([Mg_6_Al_2_(OH)_16_] (CO_3_)_3_·4H_2_O), which is a natural occurring mineral. The basic layer structure of LDHs is based on that of brucite [Mg(OH)_2_], in which Mg^2+^ is partially replaced by Al^3+^, and the positive charge is compensated by negative ions located in the interlayer space [27,28]. Taylor (1969) and Allmann (1968) first confirmed the structure and characteristics of LDHs by X-ray diffraction analysis [29,30]. The reported LDHs materials, which main laminates are composed of divalent and trivalent metal elements, mainly use Mg-Al, Ni-Al, Mn-Al, and Mg-Fe as metal laminates. Organic-modified LDHs have also been investigated [31,32,33]. It was confirmed that Mn and Fe enriched materials could oxidize As(III) to As(V) and reacted better in As(III) uptake than that of As(V) [34,35]. However, using iron and manganese simultaneously as the laminates are rarely reported, and EDTA intercalation Mn-Fe LDHs have never been reported. As a non-toxic, powerful chelating agent with abundant function groups, EDTA is widely used for heavy metals removal [36]. Many studies have shown that EDTA intercalation into LDHs can improve the adsorption efficiency [37,38] as well as retain their chelating ability [39].

The main objectives of this study were: (1) synthesis of the intercalated Mn-Fe layered double hydroxides (intercalated MF-LDHs) and characterization them with a variety of techniques; (2) systematically examination the effect of initial pH and adsorbent dosage on As(III) and As(V) adsorption onto EDTA@MF-LDHs; (3) analysis and discussing the mechanism of As(III) adsorption on EDTA@MF-LDHs.

## 2. Materials and Methods

### 2.1. Materials

All the reagents were in analytical purity and used without further purification. As(III) stock solutions and As(V) stock solutions were prepared using NaAsO_2_ and NaAsO_4_·12H_2_O, respectively. Arsenic working solutions were freshly prepared by diluting arsenic solutions with NaCl solutions which made the background ionic strength of arsenic solution to be 0.01 mol/L.

### 2.2. Preparation of Intercalated Mn-Fe LDHs

Intercalated Mn-Fe LDHs were synthesized by the co-precipitation of Mn and Fe salts at constant pH with low supersaturation. Detailed descriptions of synthesis methods are given as follows.

#### 2.2.1. Preparation of EDTA@MF-LDHs

In order to prevent the interference of carbonate, EDTA@MF-LDHs was prepared under a nitrogen atmosphere: (1) FeCl_3_·6H_2_O (4.505 g) and MnCl_2_·4H_2_O (6.583 g) were dissolved in 50 mL deoxygenated water to get the mixed metal salt solution (Sol S1); (2) NaOH (9.6 g) and EDTA·2Na (4.653 g) were dissolved in 100 mL deoxygenated water to get the base solution (Sol B1); (3) Sol B1 was added into a beaker containing 100 mL deoxygenated water until the solution pH value reached 12 ± 0.1, then Sol S1 and Sol B1 were added dropwise at the rate of keeping the reaction pH constant at 12 ± 0.1 under a nitrogen atmosphere with continuous magnetic stirring. After dropping, the mixture was stirred continuously for 10 min; (4) Then the reaction system was transferred to a water bath pot and aged for 2 h at 60 °C; (5) Finally, the product was washed 3 times by ultra-pure water using vacuum filter before it was dried at −40 ℃ in vacuum freeze dryer overnight.

#### 2.2.2. Preparation of CO_3_@MF-LDHs

CO_3_@MF-LDHs has been used as reference material to demonstrate the effect of the interlayer anion. The same quantities of FeCl_3_·6H_2_O and MnCl_2_·4H_2_O as above were dissolved in 50 mL ultra-pure water to get mixed metal salt solution (Sol S2). The mixed base solution Sol B2 was prepared from NaOH (6.4 g) and Na_2_CO_3_ (7.066 g) dissolved in 100 mL ultra-pure water. Sol B2 was dropped into a beaker containing 100 mL ultra-pure water until the solution pH value reached 12 ± 0.1, then Sol S2 and Sol B2 were added dropwise at the rate of keeping the reaction pH constant at 12 ± 0.1. The whole process was performed under magnetic stirring continuously for 10 min after the dripping process was finished. The next steps were the same as for the preparation of EDTA@MF-LDHs.

### 2.3. Characterization

Phases of the intercalated Mn-Fe LDHs composites before and after arsenic removal were identified by recording their powder X-ray diffraction patterns (XRD) under Cu Kα radiation with an X’Pert PRO diffractometer (PANalytical B.V., Almelo, The Netherlands). FT-IR spectra (Thermo Fisher Scientific lnc., Waltham, MA, USA) were recorded using a Bruker VERTEX 70 spectrometer by the KBr pellet method. The surface morphologies were examined by using a JEOL JSM-7900F field emission scanning electron microscopy (SEM) (Hitachi Ltd., Tokyo, Japan). The point of zero charge was determined by using a Zetasizer Nano JS90 (Malvern, UK.) by disperse 0.01 g material in 50 mL NaCl solution with a concentration of 0.01 mol/L, and then ultrasonic dispersion for 30 min. A Thermo Scientific ESCALAB 250Xi instrument (Thermo Fisher Scientific lnc., Waltham, MA, USA) was applied to collect the X-ray photoelectron spectra (XPS) and the results were analyzed using XPS peak fitting program (XPS Peak41) to obtain more detailed information about the adsorption mechanism.

### 2.4. Batch Experiment

Most elimination experiments were performed in 100 mL plastic centrifuge tube containing 20 mL As solution with initial concentrations 10 and 50 mg/L, and configured with NaCl (0.01 mol/L) as background solution. Then the required sorbent was dosed. These tubes were shaken at 180 rpm and kept the temperature at 25 ± 1 °C. Effect of solution initial pH (2–12) and adsorbent dose (0.1–1.5 g/L) were tested for optimizing the initial pH value and dose. Sorption kinetics were examined at varied sorption time (0–1440 min), sorption isotherm was obtained at initial As concentrations from 5 to 50 mg/L. All the samples were filtered by 0.22 µm micropore membrane and the residual As(III) and As(V) concentrations were analyzed using an inductively coupled plasma-optical emission spectrometer (ICP-OES, Optima 7000DV, Perkin-Elmer Ltd., Waltham, MA, USA).

The removal efficiency and the amount of As(III) or As(V) adsorbed on MF-LDHs were calculated by using Equations (1) and (2):

*Efficiency* (%) = (*C_o_* − *C_e_*)/*C_o_* × 100 (1)

where *C_o_* and *C_e_* are the initial As(III) or As(V) concentration (mg/L) and the residual As(III) or As(V) concentration (mg/L), respectively.

*q_t_* = (*C_o_* − *C_t_*) *V*/*W* (2)

where *C_t_*, *V*, and *M* are the As(III) or As(V) concentration (mg/L) at *t*, the solution volume (mL) and the Fe-Mn LDHs dosage (g), respectively.

## 3. Results

### 3.1. Characterization of Intercalated Mn-Fe-LDHs

#### 3.1.1. XRD

**Figure 1:**
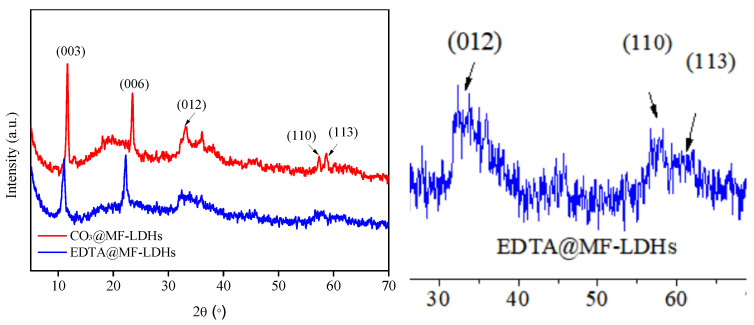
XRD pattern of CO_3_@MF-LDHs and EDTA@MF-LDHs.

The crystal structure of CO_3_@MF-LDHs and EDTA@MF-LDHs is shown in Figure 1. The XRD peaks were identified as the hydrotalcite-like phases according to the standard diffraction patterns [40,41,42]. The good multiple relationship between the typical planes of (003), (006), and (012) revealed a layered structure [43]. Similar to CO_3_@MF-LDHs, there is a diffraction peak of the (012) crystal plane indicating that the EDTA was inserted [44,45].

The main XRD indexes of CO_3_@MF-LDHs and EDTA@MF-LDHs given in Table 1 showed that the interlayer spacing of CO_3_@MF-LDHs was 0.762 nm, indicating that the interlayer anion was carbonate [42,46]. The interlayer spacing of EDTA@MF-LDHs was 0.804 nm, which was close to the interlayer spacing of EDTA Mg-Al LDH (0.8 nm) synthesized by Kameda et al. [22]. However, the spacing was smaller than that reported in the related literature (1.2–1.5 nm) [34]. It may be that the [EDTA]^4−^ was arranged in a single-layer inclined orientation between the layers. The crystallographic parameters a representing the distance between metal atoms in the adjacent hexagonal cell, and c representing the cell thickness, reflects the charge density of the laminate and are related to the size of interlayer anions. These values are approximately equal to 2d(110) and 3d(110), respectively [47]. The results show that there was a slight difference in value ***a*** between the carbonate type LDHs and the ethylenediamine tetraacetate type LDHs, indicating that there were some differences in the laminate structure and arrangement between them, which was consistent with the XRD results.

#### 3.1.2. SEM and EDS

**Figure 2:**
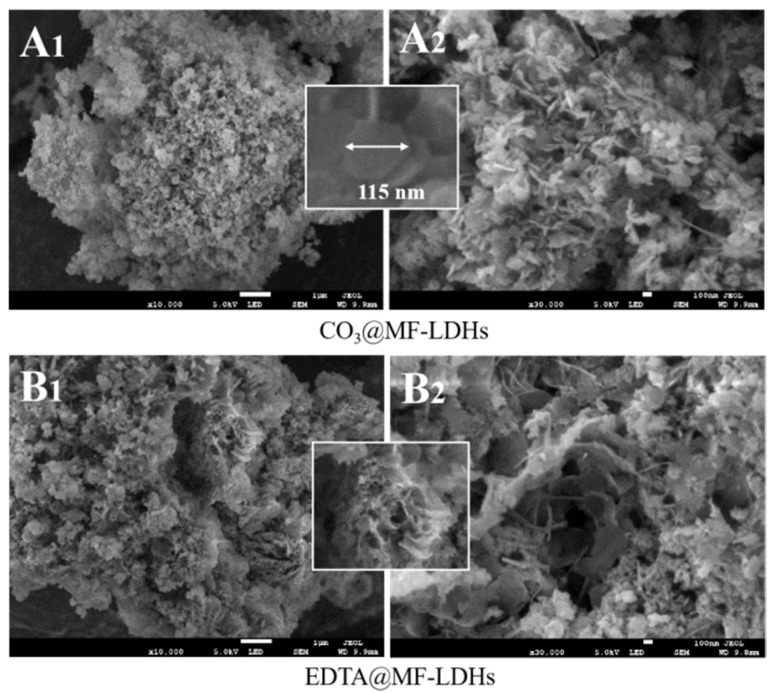
SEM analysis of CO_3_@MF-LDHs and EDTA@MF-LDHs. (Magnified both in 10,000 times (**A_1_**,**B_1_**) and 30, 000 times (**A_2_**,**B_2_**), respectively).

The surface morphologies of CO_3_@MF-LDHs and EDTA@MF-LDHs are illustrated in Figure 2. It can be seen that the surface of the samples was rough and porous, which was conducive to adsorption. As indicated in Figure 2A_1_, the CO_3_@MF-LDHs product had a typical flat hexagonal laminate morphology and obvious laminate accumulation phenomenon, which was a typical carbonate-type LDHs morphology with a particle size of 50 nm–200 nm Figure 2A_2_. The EDTA@MF-LDHs wafers with bending morphology of the laminates were prepared in the solution containing EDTA, exhibiting a rose-like agglomeration morphology on the surface of the particles with a particle size of 200 nm–500 nm (Figure 2B_1_,B_2_). This result indicated that the addition of EDTA greatly inhibited the crystal growth and induced the deformation of the laminates.

The energy dispersive X-ray spectroscopy (EDS) result (Supplementary Table S1) indicated that C, O, Fe, and Mn were detected on the surface of both CO_3_@MF-LDHs and EDTA@MF-LDHs with the Mn/Fe molar ratio of 2.04 and 1.94, respectively, closing to the theoretical value of 2.0. Compared with CO_3_@MF-LDHs, the Cl element was also detected on the surface of EDTA@MF-LDHs. The existence of the Cl element (may from MnCl_2_ and FeCl_3_) suggested the co-intercalation of Cl^−^ and [EDTA]^4−^ in the layers [48].

#### 3.1.3. FT-IR

**Figure 3:**
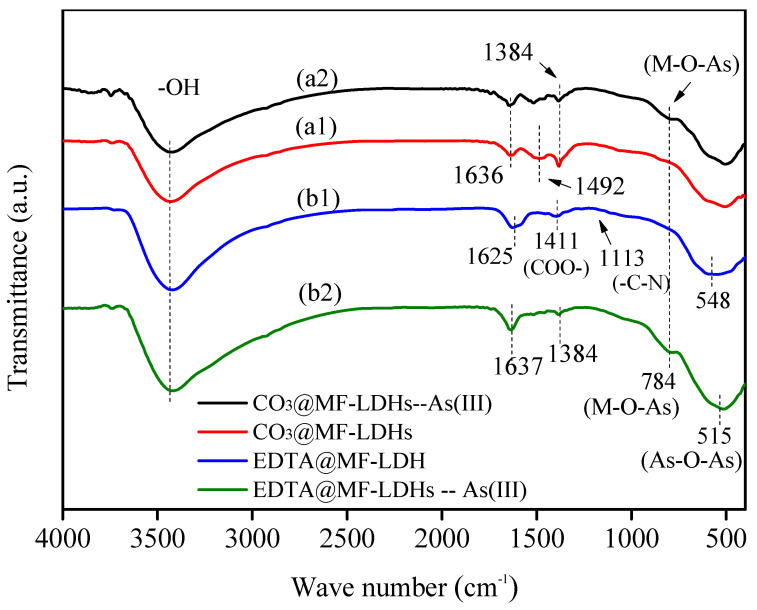
FT-IR pattern of CO_3_@MF-LDHs and EDTA@MF-LDHs before and after As(III) adsorption (a1, a2, b1, b2).

The infrared spectra of the two intercalated Mn-Fe LDHs are shown in Figure 3. Both of them had strong and wide absorption bands near 3420 cm^−1^, which were mainly caused by the superposition of -OH and laminate -OH of interlayer water molecules, indicating that there were water molecules in the interlayer. The presence of high affinity surface hydroxyl groups indicated that they could possess a good removal capacity for As(III) in water by adsorption [9,49]. For CO_3_@MF-LDHs (Figure 3, the peak at 1636 cm^−1^ was the bending vibration of interlayer water molecules, and the peak at 1384 cm was the interlayer [CO_3_]^2−^-*v*_3_ vibration mode [42,46]. The obvious shoulder peak at 1492 cm^−1^ indicated highly symmetrical and well-arranged [CO_3_]^2−^ [50]. This result confirmed the existence of carbonate between layers. The spectrum of EDTA@MF-LDHs also showed an absorption band around 1600 cm^−^^1^ (at 1625 cm^−^^1^), but its intensity changed in comparison with the former, which might be the superposition peak of the interlayer water molecule and the C=O functional group in the carboxyl group. The weak peaks at 1411 cm^−^^1^ and 1113 cm^−^^1^ corresponded to the asymmetric peak of COO- and the characteristic absorption peak of -C-N group [37], indicating the existence of [EDTA]^4−^. The acromion of the two samples in the low-frequency region (500–800 cm^−^^1^) were assigned to M–O vibrations and M–O–H bending vibration (M = Mn, Fe) [51,52,53], suggesting the presence of layered metal hydroxide (Figure 3). The results showed that the Mn-Fe LDHs with different morphology were successfully synthesized by changing the interlayer anion.

#### 3.1.4. Point of Zero Charge

**Figure 4:**
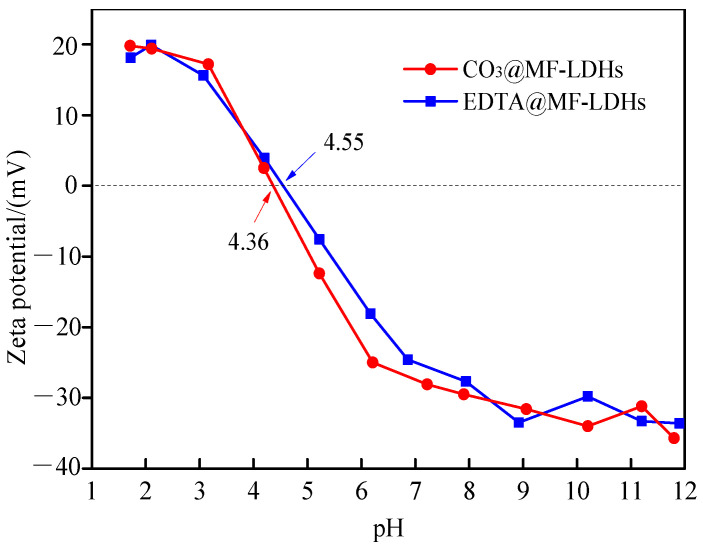
Zeta potentials of CO_3_@MF-LDHs and EDTA@MF-LDH.

The zeta potential versus pH for CO_3_@MF-LDHs and EDTA@MF-LDHs are shown in Figure 4. With the increase of pH from 1.7 to 11.9, the zeta potential of both materials decreased from around 20 mV to around −35 mV, that is, the surface charge of the material changed from positive to negative. The zero potential (pH_pzc_) values of the two materials were 4.36 and 4.55, respectively. They were negatively charged at circumneutral pH, indicating the existence of the surface hydroxyl groups.

### 3.2. Sorption Performance

#### 3.2.1. Effect of pH

**Figure 5:**
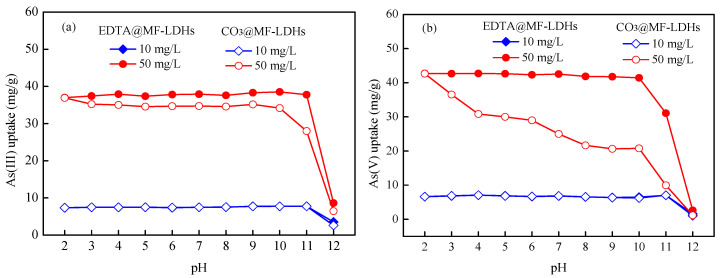
Effect of initial pH on As(III) and As(V) removal by CO_3_@MF-LDHs and EDTA@MF-LDHs. (Initial concentration 10 and 50 mg/L; dose 1.2 g/L; and temperature 25 ± 1 °C). (**a**) As(III) uptake on CO_3_@MF-LDHs and EDTA@MF-LDHs. (**b**) As(V) uptake on CO_3_@MF-LDHs and EDTA@MF-LDHs.

Initial solution pH is an important parameter in the adsorption process, which affects not only the existing forms of ions in the solution, but also the charged properties of the adsorbent surface. The residual concentrations of As(III) and As(V) stock solution without sorbents did not decrease significantly (Supplementary Figure S1), i.e., no obvious hydrolytic precipitation could be observed at pH ≤ 12. Thus, the As(III) and As(V) sorption experiment was done in the pH range of 2.0–12.0. The effects of initial pH on As(III) and As(V) removal by CO_3_@MF-LDHs and EDTA@MF-LDHs are shown in Figure 5. For 50 mg/L As(III), the As(III) removal by CO_3_@MF-LDHs was approximately constant in the pH range of 3.0–9.0 and decreased at pH > 9. The minimum adsorption capacity was observed at pH 12. The As(III) removal by EDTA@MF-LDHs was basically undisturbed when solution initial pH varied from 2 to 10; but at pH > 10, the adsorption capacity decreased sharply. At pH < 9, As(III) existed in non-ionic form of H_3_AsO_3_^0^. There was no electrostatic interaction between the surface of the material and the adsorbate. At pH > 9, As(III) existed mainly in the form of [AsO_3_]^3–^ (Supplementary Figure S2), the negatively charged adsorbents (Figure 4) restricted the approach of As(III) due to the repulsive force [54,55]. Thus, the removal of As(III) in the pH range of 2 to 10 was mainly attributed to the cooperation between the functional groups on the surface of the adsorbent and the surface complexing of As(III) [56,57]. 

The adsorption capacities for 50 mg/L As(V) on CO_3_@MF-LDHs and EDTA@MF-LDHs were significantly different in different pH ranges. The CO_3_@MF-LDHs showed a strong pH dependence, consistent with the results reported recently [58,59]. With the pH increase from 2 to 12, the adsorption capacity of As(V) on CO_3_@MF-LDHs decreased continuously from 42.66 mg/g to 1.19 mg/g. The influence of the pH value was related to two factors, i.e., the charge on the surface of the adsorbent and the existing form of As(V) in solutions of different acidities. The dissociation of arsenate acid in solution is as follows [1]:

Step one: H_3_AsO_4_→H_2_AsO_4_^−^ + H^+^ , p*K*_a1_ = 2.1;

Step two: H_2_AsO_4_^−^→HASO42^−^ + H^+^ , p*K*_a2_ = 6.7;

Step three: HAsO_4_^2−^→ AsO_4_^3−^ + H^+^ , p*K*_a3_ = 11.2;

The As(V) in the solution mainly existed in the form of H_2_AsO_4_^−^, [HAsO_4_]^2^^−^, and [AsO_4_]^3^^−^ [10,19]. With the pH increase from 2 to 12, the negative charge on the surface of the adsorbent increased, leading to a continuous decrease in the As(V) adsorption capacity due to repulsive force. However, the As(V) removal efficiency of EDTA@MF-LDHs only had a slight change due to the hydrophobic surface of LDHs intercalated by EDTA and the protective layer on the surface of the material, which could neutralize the influence of the solution pH change. The adsorption capacities decreased only at pH = 12 and initial concentration of 10 mg/L As(III) and As(V) because there were enough binding sites on the sorbents due to the relatively low concentrations of the solutions. Thus, the subsequent experiments were carried out at pH = 7.

#### 3.2.2. Effect of Dose

**Figure 6:**
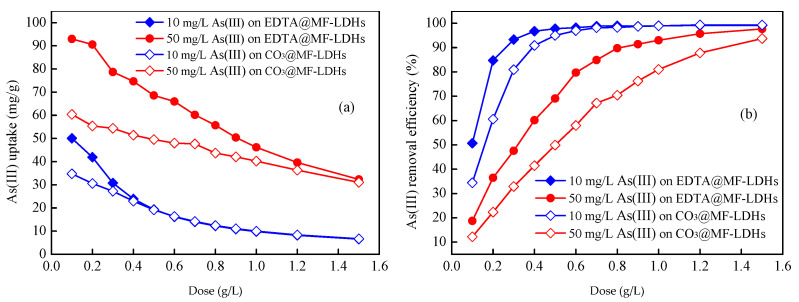

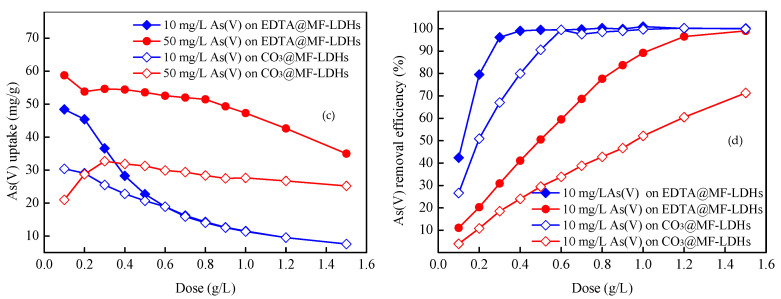
Effect of adsorber dose on As(III) and As(V) removal by CO_3_@MF-LDHs and EDTA@MF-LDHs. (CO_3_@MF-LDHs, EDTA@MF-LDHs dose from 0.1 to 1.5 g/L; As initial concentration are 10 and 50 mg/L; initial pH 7.0 ± 0.2; temperature 25 ± 1 °C). (**a**) As(III) uptake on CO_3_@MF-LDHs and EDTA@MF-LDHs. (**b**) As(III) removal efficiency on CO_3_@MF-LDHs and EDTA@MF-LDHs. (**c**) As(V) uptake on CO_3_@MF-LDHs and EDTA@MF-LDHs. (**d**) As(V) removal efficiency on CO_3_@MF-LDHs and EDTA@MF-LDHs..

The effect of adsorbent dosage on arsenic removal from solution are shown in Figure 6a–d. For the As(III) and As(V) solution both in 10 mg/L, the unit As(III) uptake decreased from 50.10 mg/g to 6.54 mg/g and unit As(V) uptake decreased from 48.41 mg/g to 7.61 mg/g with increasing the EDTA@MF-LDHs dose from 0.1 g/L to 1.5 g/L. Correspondingly, the elimination efficiency of As(III) increased from 50.64% to 97.78% and of As(V) from 42.39% to 99.99%, continuously. For the As(III) and As(V) solution both in 50 mg/L, the unit uptake decreased from 93.00 mg/g to 32.33 mg/g (As(III)) and from 50.50 mg/g to 35.20 mg/g (As(V)), respectively, while the elimination efficiency increased from 18.73% to 97.67% (As(III)) and from 9.53% to 99.58% (As(V)), respectively. Similarly, with the increase of the CO_3_@MF-LDHs dose, the unit As(III) and As(V) uptake decreased while the elimination efficiency increased. The increase of the removal percentage with EDTA@MF-LDHs dose was attributed to surface area increase of EDTA@MF-LDHs and availability sites. The dose of 0.5 g/L was applied in the subsequent experiments.

Preliminary studies revealed that the prepared EDTA@MF-LDHs adsorbent was more effective for both As(III) and As(V) removal than CO_3_@MF-LDHs. Moreover, much more As(III) than As(V) could be adsorbed onto EDTA@MF-LDHs under the same experimental conditions. Thus, EDTA@MF-LDHs was selected for further As(III) removal study.

#### 3.2.3. Sorption Kinetics

**Figure 7:**
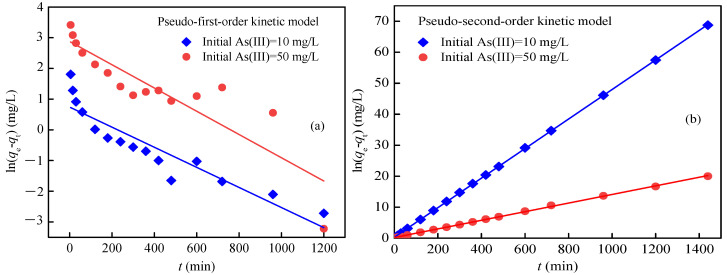
Kinetics isotherm for As(III) removal by EDTA@MF-LDHs.(pH 7.0 ± 0.2; temperature 25 °C). (**a**) Pseudo-first-order kinetic model. (**b**) Pseudo-second-order kinetic model.

A series of kinetic models were used to fit the experimental data to investigate the sorption mechanism demonstrated. The pseudo-first-order and pseudo-second-order kinetic models are given in Equations (3) and (4):

(3) $$ln(q_{e}-q_{t})=\ln q_{e}-K_{1t}$$

(4) $$\frac{t}{q_{t}}=\frac{1}{k_{2}q_{e}^{2}}+\frac{t}{q_{e}}$$

where *k*_1_ and *k*_2_ are the pseudo-first-order and pseudo-second-order constants (g/(mg·min)), respectively, *q*_t_ is the As(III) uptake at time *t* (mg/g) and *q*_e_ is the As(III) uptake at equilibrium (mg/g). The kinetics parameters obtained were summarized in Table 2 and Supplementary Table S2. The experimental data could be fitted to the pseudo-second-order kinetic model with *R*^2^ = 1.0000 for 10 mg/L As(III) and *R*^2^ = 0.9994 for 50 mg/L As(III), which was better than the fitting of the pseudo-first-order kinetic model (*R*^2^ = 0.8428 and 0.7649) (Figure 7), Elovich and Morris intra-particle diffusion models (Supplementary Figure S3). For the initial As(III) concentration of 10 and 50 mg/L, the calculated As(III) uptakes at equilibrium (*q*_e_) of 20.96 mg/g and 71.94 mg/g were well in agreement with the measured data of 20.95 mg/g and 71.98 mg/g, respectively. The low *K*_2_ value implied that the adsorption rate decreased with the increase in contact time and the adsorption rates were proportional to the number of adsorption sites. Therefore, the adsorption rates were controlled by chemisorption involving valence forces [60] and the As(III) adsorption capacity of EDTA@MF-LDHs depended mainly on the active sites on the material surface. On the other hand, according to the effect of contact time on As(III) adsorption onto EDTA@MF-LDHs shown in Supplementary Figure S3a, specific adsorption occurring between As(III) and EDTA@MF-LDHs, because adsorption solely due to electrostatic processes was usually in the order of seconds but not hours [58].

#### 3.2.4. Sorption Isotherms

**Figure 8:**
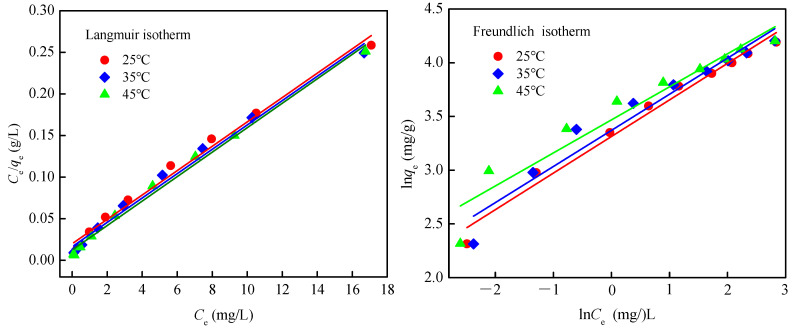
Isotherm for As(III) removal by EDTA@MF-LDHs. (initial concentration 5–50 mg/L; pH 7.0 ± 0.2; temperature 25–45 °C).

The adsorption results were fitted with the Langmuir and Freundlich isotherm models as given in Equations (5) and (6), respectively:

(5) $$C_{e}/q_{e}=1/(q_{m}\cdot K_{L})+C_{e}/q_{m}$$

(6) $$\ln q_{e}=\ln K_{F}+1/n\ln C_{e}$$

where *q*_e_ is the amount (mg/g) of As(III) adsorbed at equilibrium, *C*_e_ is the equilibrium As(III) concentration (mg/L) in water samples, *K*_L_ and *q*_max_ (maximum adsorption capability) are the Langmuir constants, and *K*_F_ and *n* are the Freundlich constants. The sorption isotherm fittings and the corresponding fitting parameters are reported in Figure 8 and Table 3, respectively. As can be observed, both isothermal models could well describe the isothermal adsorption process of As(III) onto EDTA@MF-LDHs with high fitted correlation coefficient (*R*^2^ > 0.92). The *R*^2^ value of Freundlich isotherm model was slightly higher and the adsorption of As(III) onto EDTA@MF-LDHs belonged to the multi-layer adsorption on the inhomogeneous surface [1]. The value of parameter 1/*n* can be used to characterize the nonlinear growth trend of adsorption [61]. The 1/*n* value between 0.1 and 0.5 indicated that As(III) was easy adsorbed by EDTA@MF-LDHs. The maximum As(III) adsorption capacity (*q*_m_) of EDTA@MF-LDHs was determined from the Langmuir model to be 68.49 mg/g, and the reletive adsorption constant *K*_L_ from 0.734 to 1.186, both the *q*_m_ and *K*_L_ are limilar to or slightly higher than many reported meterials, such as ferrous hydroxide colloids (FHC) [1], Mn-Fe-LDH [59], Fe_3_O_4_/Cu(OH)_2_ [62], aspartic acid intercalated LDH [63] and magnetic Fe_3_O_4_@CuO nanocomposite [64], listed as in Table 4. This means that EDTA@MF-LDHs can be suitable adsorbent for the removal of As(III) from aqueous solution. An extremely high As(III) adsorption maxima of 0.58 mol As/mol Fe (778 mg/g) for ferryhydrite (Raven et al., 1998) could be an experimental anomaly attributable to the high initial As(III) solution concentration of 26.7 mmol/L (2000 mg/L) used in their studies [65].

#### 3.2.5. Removal Mechanisms

To insight into the sorption mechanism of As(III) by EDTA@MF-LDHs, a matrix of complimentary analyses were performed. First, the FT-IR spectra of CO_3_@MF-LDHs and EDTA@MF-LDHs before and after sorption are exhibited in Figure 3. After CO_3_@MF-LDHs adsorbed As(Ⅲ) (a2), the vibration peak of CO_3_^2−^ (1384 cm^−1^) did not disappear and the peak position almost did not shift. This may be due to the strong binding force between CO_3_^2−^ and the metal laminate, which makes it difficult to exchange with the anions in the aqueous solution, so the vibration peak of carbonate (1384 cm^−1^) still exists. After As(III) adsorption on EDTA@MF-LDHs (b2), the adsorbent still showed a strong and wide diffraction peak of -OH in the range of 3200–3500 cm^−1^, but it shifted slightly to low frequency, which might be attributed to the electrostatic attraction between the protonated oxygen-containing groups and anions [59]. There was a strong shoulder peak at 784 cm^−1^ after As(III) adsorption, which was inferred as the Fe-As-O and Mn-As-O bonds, indicating that As(III) was effectively adsorbed to form an inner complex on the surface of the adsorbent. In addition, the characteristic peaks at 548 cm^-1^, corresponding to the metal lattice, shifted to a low frequency 515 cm^−1^ after As(III) adsorption. Therefore, Fe-O and Mn-O bonds were also involved in the adsorption process with a new characteristic peak of As-O-As appeared. The peak position of the C=O functional bonds in the carboxyl group moved from 1625 cm^−^^1^ to 1636 cm^-1^ and the peak at 1113 cm^−^^1^ disappeared after As(III) adsorption, which indicated that [EDTA]^4^^−^ participated in the adsorption process through C=O and C-N groups [38,66]. After As(III) adsorption by EDTA@MF-LDHs, there was an obvious characteristic peak at 1384 cm^−^^1^ corresponding to [CO_3_]^2^^−^, which might be introduced in the adsorption process.

**Figure 9:**
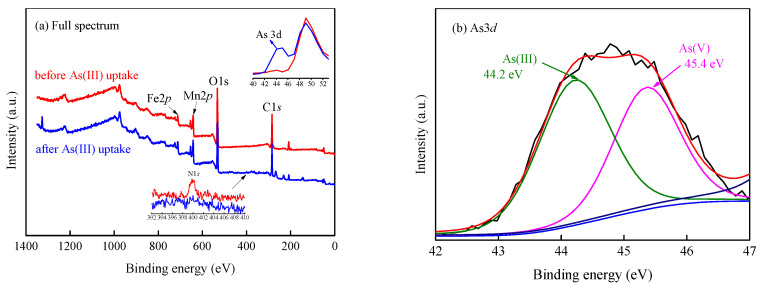

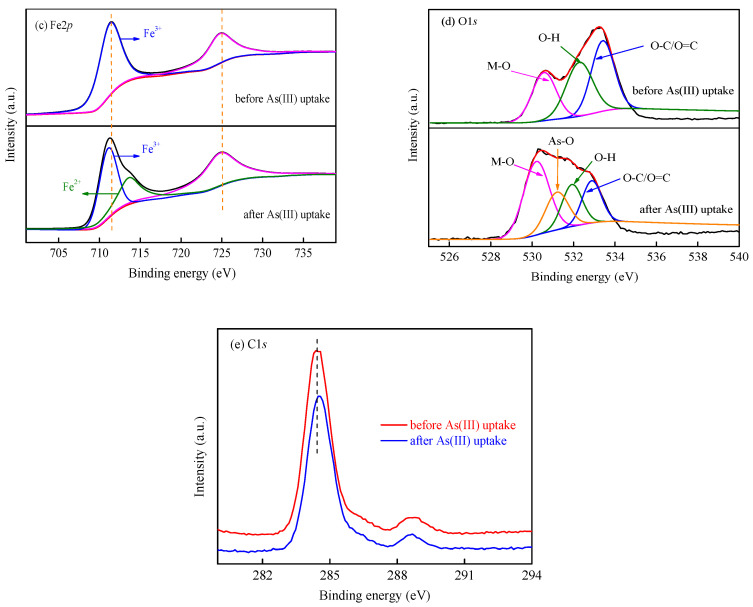
XPS spectral of (**a**) Full spectrum, (**b**) As3*d*, (**c**) Fe2*p,* (**d**) O1*s,* and (**e**) C1*s* for EDTA@MF-LDHs before and after As(III) adsorption (initial concentration 50 mg/L).

The reported studies have confirmed that the test conditions of XPS (heating and vacuum) have no influence on the state of As [24,67,68]. The chemical compositions of EDTA@MF-LDHs before and after As(III) adsorption were further probed by XPS analyses in this stdudy. The main peaks of Fe2*p*, Mn2*p*, C1*s*, O1*s*, and N1*s* were detected in the XPS full spectra before and after adsorption (Figure 9). The existence of the N1*s* peak further proved the existence of [EDTA]^4^^−^. The As3*d* peak (44.1–45.6 eV) in the spectrum of the sample after As(III) adsorption confirmed that As(III) was adsorbed on EDTA@MF-LDHs. In order to reveal more accurately the chemical forms of EDTA@MF-LDHs after As(III) adsorption, the high-resolution spectra of As, Fe, O, and C before and after adsorption were further analyzed with the corresponding peak fitting as shown in Figure 9b–e. It was found that there were both trivalent arsenic (44.2 eV) and pentavalent arsenic (45.4 eV) peaks in the As3*d* spectrum after As(III) adsorption, indicating that As(III) was partially oxidized to As(V) during the adsorption (Figure 9b).

As shown in Figure 9c, the binding energy of Fe2*p* shifted from 711.11 eV to 711.19 eV after As(III) adsorption. The shifting of binding energy indicated that the physical and chemical environment around the Fe element in EDTA@MF-LDHs had changed. The co-existence of Fe(III) and Fe(II) after As(III) adsorption might be related to the redox interaction between Fe(III) and As(III). The spectra of O1*s* showed the additional As-O peaks and the peak area assigned to C-O/C=O and O-H decreased after As(III) adsorption (Figure 9d), reflecting a decrease in their quantity. After As(III) adsorption, the characteristic peaks of the C1*s* spectra shifted from 284.44 eV to 284.54 eV and the peak intensities decreased noticeably (Figure 9e). We speculated that the oxidized As(V) was exchanged with interlayer [EDTA]^4^^−^ and surface hydroxyl functional groups.

**Figure 10:**
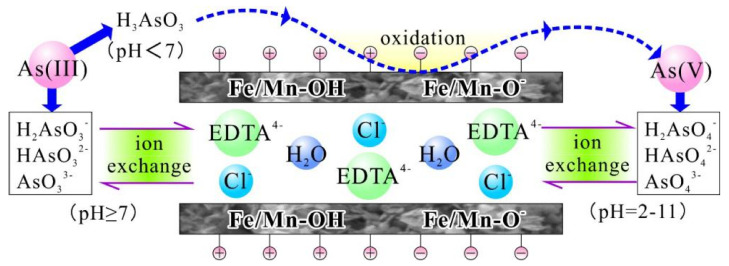
Mechanism of As(III) elimination by EDTA@MF-LDHs.

Based on the above analysis, the mechanism of As(III) adsorption onto EDTA@MF-LDHs was a complicated process including surface complexation, redox, and ion exchange (Figure 10). In the solution of pH = 7, As(III) existed mainly as a neutral molecule (H_3_AsO_3_), which could exchange ligands with the plentiful hydroxyl groups on the surface of the metal laminates, and then As(III) was oxidized to As(V) by Fe(III) around pH = 4.55~12, Mn(II)/Fe(III)–OH was reduced to 2≡Mn(II)/Fe(II)–O^−^ (Figure 9d), so it is negative charge on the EDTA@MF-LDHs surface (Figure 4), Mn(II) is the structural carrier of bimetallic hydroxide. The oxidizing reaction process as follows:

(7) $$2\;\equiv \;Mn/Fe\;-\;OH\;+\;H_{3}AsO_{3}\;+\;H_{2}O\to 2\;\equiv \;Mn/Fe\;-\;OH^{+}\;+\;HAsO_{4}^{-}\;+\;H_{2}O$$

(8) $$2\;\equiv \;Mn/Fe\;-\;O\;+\;H_{3}AsO_{3}\;+\;H_{2}O\to 2\;\equiv \;Mn/Fe\;-\;O^{+}\;+\;HAsO_{4}^{-}\;+\;H_{2}O$$

Meanwhile, As(V) could be further exchanged with interlayer anions including Cl^−^ and [EDTA]^4^^−^. The ion exchange between arsenate ions and Cl^−^ could be distinctly confirmed by the EDS result (Supplementary Table S3). Cl^−^ could not be detected on the surface of the adsorbent after adsorption.

#### 3.2.6. Material Stability Test

**Figure 11:**
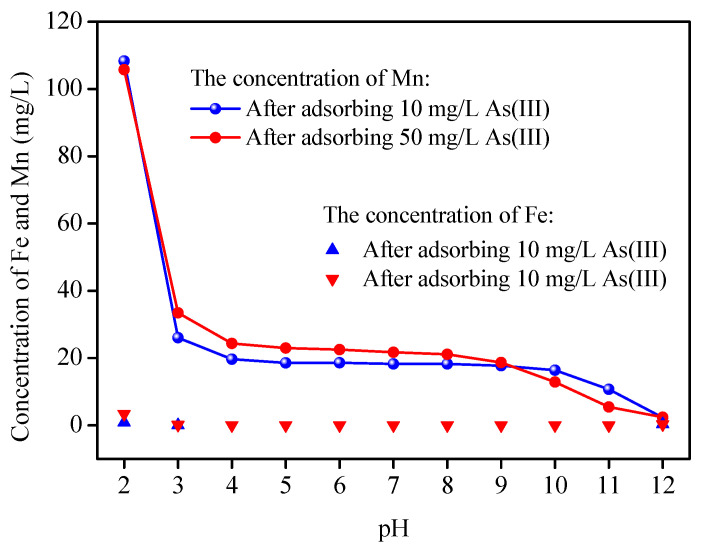
The stability test of EDTA@MF-LDH after As(III) adsorption. (adsorbent dose 0.5 g/L; pH 7.0 ± 0.2; temperature 25 ± 1 ℃).

In this experiment, the chemical stability of EDTA@MF-LDH was discussed. The results showed that nearly no iron ion was detected in the solution after As (III) adsorption, but manganese ion was dissolved, and at pH 11, the concentration of manganese ion is below 10 mg/L. It can also be seen that the concentration of As(III) has no obvious effect on the dissolution of iron and manganese. The change trend of manganese concentration in solution corresponds to that of As(III) adsorption on EDTA@MF-LDH (Figure 5). The specific results are shown in Figure 11.

## 4. Conclusions

A novel and highly efficient adsorbent, EDTA@MF-LDHs with the lamellar structure, was first designed for the application of As(III) removal. Compared with CO_3_@MF-LDHs, EDTA@MF-LDHs showed a higher interlayer spacing 0.804 nm, which was helpful for As(III) to enter the interlayer of LDHs for ion exchange. EDTA@MF-LDHs had a high adsorption capacity for As(III) in a wide pH range of 2 to 11. When the dosage of EDTA@MF-LDHs was 0.5 g/L, the removal efficiency of As(III) (10 mg/L) was maintained at a level above 97%. The adsorption capacities of EDTA@MF-LDHs towards As(III) were 68.49 mg/g determined from the Langmuir model, which is similar to or slightly higher than many reported adsorbents (Table 4). We also found that As(III) could be oxidized to As(V) by EDTA@MF-LDHs directly without adding any oxidant. This reduced the pollution toxicity as well as promoted ion exchange. In the EDTA@MF-LDHs-As(III) systems, the As(III) adsorption removal mechanism includes surface complexation, redox, and ion exchange. Remarkably, this work provides a strategy for As(III) removal adsorbent MF-LDHs (metal laminates were derived from transition metals) synthesis. Meanwhile, it also provides a clearer understanding of adsorption mechanisms which would be beneficial for further design of related adsorbent.

## Figures and Tables

**Table 1:** XRD index of CO_3_@MF-LDHs and EDTA@MF-LDHs (nm).

Diffraction Index	d(003)	d(006)	d(012)	d(110)	*a* = 2d(110)	*c* = 3d(003)
CO_3_@MF-LDHs	0.762	0.381	0.271	0.161	0.322	2.286
EDTA@MF-LDHs	0.804	0.400	0.276	0.162	0.324	2.412

**Table 2:** Kinetics parameter for As(III) adsorption onto EDTA@MF-LDHs.

Initial As(III) Concentration (mg/L)	Pseudo-First-Order Constant			Pseudo-Second-Order Constant			
	*K*_1_ (/min)	*q*_e_ (mg/g)	*R* ^2^	*k*_2_ (g/(mg·min))	*h* (g/(mg·min))	*q*_e_ (mg/g)	*R* ^2^
10	0.003	2.010	0.843	0.008	3.279	20.960	1.000
50	0.004	17.780	0.765	0.001	4.684	71.940	0.999

**Table 3:** Isotherm parameter for As(III) adsorption onto EDTA@MF-LDHs.

Temperature (°C)	Langmuir Constant				Freundlich Constant		
	*q*_m_ (mg/g)	*K*_L_ (L/mg)	*R*_L_	*R* ^2^	*K*_F_ (mg^1−1/n^·L^1/n^/g)	1*/n*	*R* ^2^
25	68.49	0.734	0.027–0.214	0.986	27.481	0.341	0.979
35	68.49	0.896	0.022–0.183	0.989	29.093	0.336	0.942
45	68.03	1.186	0.017–0.144	0.992	28.413	0.308	0.917

**Table 4:** Comparison of arsenic adsorption capacity between EDTA@MF-LDHs and other adsorbents.

Adsorbents	Temperature (°C)	Concentration of As(III) (mg/L)	*Q*_max_ (mg/g)	*K*_L_ (L/mg)	Source
Ferryhydrite	-	2000	778	-	[65]
Ferryhydrite	25	100	122.63	0.302	[1]
Mn–Fe-LDH	45	100	113.12	-	[59]
Fe_3_O_4_/Cu(OH)_2_	25	85	37.97	-	[62]
Mg_7_Zn_7_Fe_4_-Asp-LDH	25	100	94.81	0.2664	[63]
Mg_7_Zn_1_Fe_4_-Phe-LDH	25	100	58.09	0.0988	
Fe_3_O_4_@CuO	25	75	50.58	0.058	[64]
Fe@Cu&GO	25		70.36	0.182	
MgAl-MoS_4_-LDH	25	400	99	-	[66]
EDTA@MF-LDHs	25	50	68.49	0.734	This study
	35		68.49	0.896	
	45		68.03	1.186	

Note:“-” means no data.

## Supplementary Materials

The following are available online at https://www.mdpi.com/1660-4601/17/24/9341/s1, Figure S1: Effect of pH on the residual concentration of 10 and 50 mg/L As(III/V) stock solution (temperature 25 ± 1 °C), Figure S2: As(III) species with the change of the solution pH (As(III) = 50 mg/L; chloride ion strength of 0.01 mol/L), Figure S3: Effect of contact time on As(III) adsorption onto EDTA@MF-LDHs. (a): Fitting with Elovich kinetic model; (b): Fitting with the Morris intra-particle diffusion model; (c): Initial As(III) concentration 10 and 50 mg/L; sorbent dose 0.5 g/L; pH 7.0 ± 0.2; temperature 25 ± 1 °C, Table S1: EDS analysis of EDTA@MF-LDHs before and after As(III) adsorption (%), Table S2 Kinetics parameter for As(III) adsorption onto EDTA@MF-LDHs.
