# Supplementary material for: Mn-Fe Layered Double Hydroxide Intercalated with Ethylene-Diaminetetraacetate Anion: Synthesis and Removal of As(III) from Aqueous Solution around pH 2–11
Source: Int J Environ Res Public Health. 2020 Dec 14;17(24):9341. doi: 10.3390/ijerph17249341 (PMC7764843; doi:10.3390/ijerph17249341)
Supplement: Supplementary file 1 [file ijerph-17-09341-s001.pdf]

Supplementary Material

Mn-Fe layered double hydroxide intercalated with ethylene-diaminetetraacetate anion: Synthesis and application for removal of As(III) from aqueous solution around pH 2-11

Guifeng Liu[1], Zongqiang Zhu[1,2,*], Ningning Zhao[1], Yali Fang[1], Yingying Gao[1], Yinian Zhu[1,*], Lihao Zhang[1]

[1] Guangxi Key Laboratory of Environmental Pollution Control Theory and Technology, Guilin University of Technology, Guilin 541004, China. liuguifengmail@163.com (L.G.); zhuzongqiang@glut.edu.cn (Z.Z.); 18835175975@163.com (N.Z.); fangyali0610@163.com (F.Y.); gyy88882020@163.com (G.Y.); zhuyinian@glut.edu.cn (Y.Z.); lhzhang@glut.edu.cn (Z.L.)

[2] State Key Laboratory of Environmental Aquatic Chemistry, Research Center for Eco-Environmental Sciences Chinese Academy of Sciences, Beijing 100085, China

* Corresponding authors. emails: zhuzongqiang@glut.edu.cn (Z.Z.) and zhuyinian@glut.edu.cn (Y.Z.)

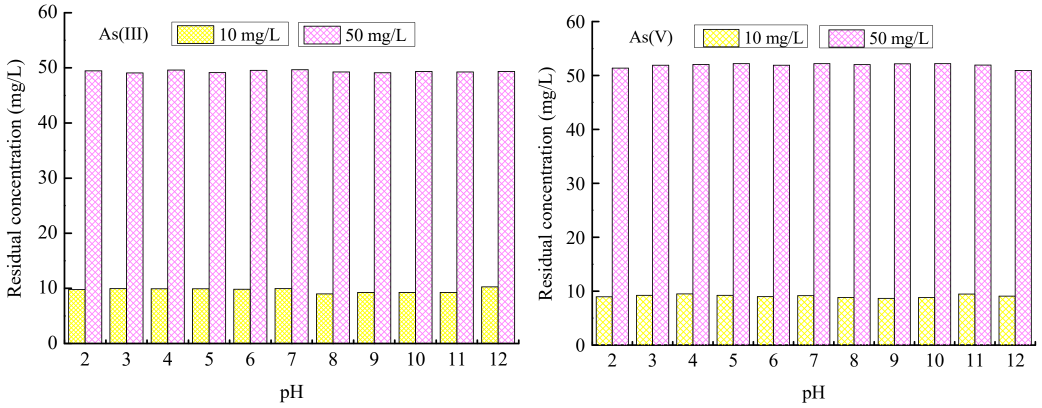

Figure S1. Effect of pH on the residual concentration of 10 and 50 mg/L As(III/V) stock solution (temperature 25±1°C).

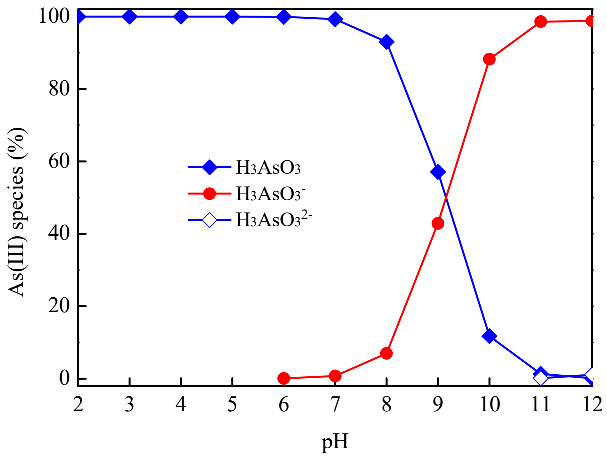

Figure S2. As(III) species with the change of the solution pH (As(III)=50 mg/L; chloride ion strength of 0.01 mol/L).

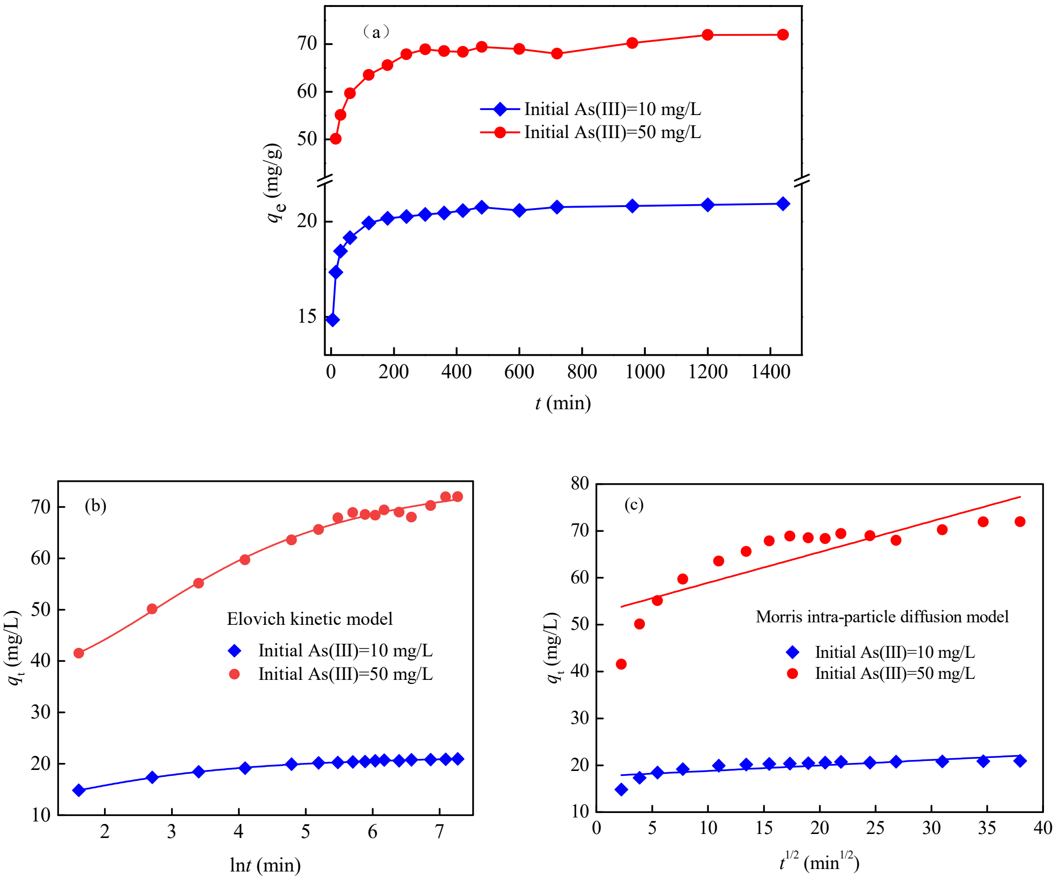

Figure S3. Effect of contact time on As(III) adsorption onto EDTA@MF-LDHs (a); Fitting with Elovich kinetic model (b); Fitting with the Morris intra-particle diffusion model (c). (Initial As(III) concentration 10 and 50 mg/L; sorbent dose 0.5 g/L; pH 7.0±0.2; temperature 25±1°C)

Table S1. EDS analysis of EDTA@MF-LDHs before and after As(III) adsorption (%).

EDTA@MF-LDHs	elements	region 1	region 2	region 3	average
before	C	29.58	31.40	31.04	30.67
	O	21.66	16.38	12.37	16.80
	Cl	2.93	2.69	2.63	2.75
	Mn	29.54	32.57	35.92	32.68
	Fe	16.30	16.96	18.04	17.10
after	C	7.26	9.66	16.94	11.29
	O	33.27	37.31	36.95	35.84
	Cl	–	–	–	–
	Mn	31.86	24.97	23.21	26.68
	Fe	19.10	18.56	14.47	17.38

Note:-, not detected

Table S2. Kinetics parameter for As(III) adsorption onto EDTA@MF-LDHs

Elovich constant			
**Initial As(III) concentration (mg/L)**	**a_e (mg/g·min)**	**b_e (g/mg)**	**R^2**
10	$5.10*10^{-6}$	1.053	0.998
50	0.007	0.195	0.990

Morris intra-particle diffusion constant (fitting data with single straight line)		
**Initial As(III) concentration (mg/L)**	**k_d (mg/(g·min$^{1/2}$))**	**R^2**
10	0.116	0.563
50	0.656	0.665
